# West Nile Virus Antibody Prevalence in Wild Mammals, Southern Wisconsin

**DOI:** 10.3201/eid1212.060173

**Published:** 2006-12

**Authors:** Douglas E. Docherty, Michael D. Samuel, Cherrie A. Nolden, Kristina F. Egstad, Kathryn M. Griffin

**Affiliations:** * US Geological Survey, Madison, Wisconsin, USA:; † University of Wisconsin, Madison, Wisconsin, USA

**Keywords:** Wisconsin, serosurvey, West Nile virus, wild mammals, dispatch

## Abstract

Twenty percent prevalence of West Nile virus antibody was found in free-ranging medium-sized Wisconsin mammals. No significant differences were noted in antibody prevalence with regard to sex, age, month of collection, or species. Our results suggest a similar route of infection in these mammals.

In 1999, West Nile virus (WNV) was detected for the first time in the United States in dead American crows (*Corvus brachyrhynchos*), and a disease surveillance system that used dead crows was established (*1**,**2*). Serologic surveys to determine the prevalence of WNV exposure in free-ranging mammals (*3**–**6*) are relatively rare. Although WNV can infect a wide range of vertebrates, mammals are assumed to be dead-end hosts (*7*). We report the results of a 2003–2004 WNV serosurvey in medium-sized mammals from south-central Wisconsin.

## The Study

We obtained samples from a part of south-central Wisconsin (Dane and Iowa Counties) recently identified as an area where white-tailed deer (Odocoileus virginianus) had chronic wasting disease infection (*8*). Medium-sized free-ranging mammals were collected as part of a larger study to evaluate the potential for transmission of chronic wasting disease from infected white-tailed deer carcasses to scavenging mammals. A total of 228 medium-sized mammal carcasses, consisting of 78 raccoons (Procyon lotor), 71 Virginia opossums (Didelphis virginiana), 59 coyotes (Canis latrans), 7 red foxes (Vulpes vulpes), 6 striped skunks (Mephitis mephitis), 5 feral cats (Felis catus), and 2 badgers (Taxidea taxus), were obtained by trapping, shooting, or collecting fresh road kills during October 2003 through April 2004. These animals were collected in rural areas consisting of small woodlots, agricultural fields, and roadsides.

Blood samples from the carcasses were collected by absorbtion into Nobuto strips (Toyo Roshi Kaisha, Ltd, Tokyo, Japan), labeled, air dried, and stored at ambient temperature until submitted to the National Wildlife Health Center (NWHC). A 1:20 serum dilution was prepared in the laboratory by following the manufacturer's instructions for extraction from the Nobuto strip. The dilution was stored at 0°C until it was tested.

Before testing, serum samples were heat inactivated (56°C for 30 min) to eliminate any nonspecific virus inhibitors. Serum controls were included for each sample to determine whether any individual serum sample was toxic to the cell culture used. The samples were screened for WNV antibody against 100 PFU by using the plaque reduction neutralization test (PRNT) (*9*). The WNV used was isolated by NWHC in September 1999 from the spinal cord, sciatic nerve, and brain pool of an American crow found dead in the state of New York (strain NY99–35261–11). Serum samples were considered to be positive for flavivirus antibody if they neutralized >50% of the WNV test dose at a serum dilution >1:40. Positive serum samples were subsequently titered by PRNT (*9*) against both WNV and Saint Louis encephalitis virus (SLEV) to determine antibody titer and specificity. The SLEV strain (TBH-28 ASFL) was obtained from the Centers for Disease Control and Prevention, Atlanta, Georgia. Serum antibody titers were determined by attempting to neutralize WNV and SLEV using 2-fold serial dilutions ranging from 1:20 to 1:2,560. The serum titer endpoint was considered to be that dilution >1:40 still capable of neutralizing >90% of the virus test dose. The antibody titer of each serum against the 2 viruses was compared. Serum samples were considered positive for WNV antibody if the titer was >4-fold more than the serum titer against SLEV. If a <2-fold SLEV and WNV titer difference was noted, the serum antibody was considered to be due to exposure to a previously described or not yet recognized flavivirus.

## Conclusions

In 2001 the Wisconsin Department of Health and Family Services (DHFS) reported the first isolation of WNV from a crow (DHFS, unpub. data), and surveillance for the virus was initiated throughout Wisconsin. By 2003, WNV was detected throughout Wisconsin (including our sampling area); most positive corvid cases coincided with our sampling period from late summer to fall. The Wisconsin Department of Natural Resources reported (http://www.dnr.state.wi.us) that WNV had been detected in 145 (48%) of 301 dead American crows and 17 (22%) of 77 dead blue jays (Cyanocitta cristata) tested. Most of these positive avian cases were detected from mid-August through October. WNV was also detected in 70 of 72 Wisconsin counties, including the 2 in our study.

Our data indicate that the mammals tested in 2003 and 2004 were more likely to be exposed to WNV than to other flaviviruses. Of the 228 medium-sized mammals tested, 70 (31%) (Table) had flavivirus antibody, with specific WNV antibody in 46 (66%) of 70. Because the numbers of samples were insufficient, we could not evaluate WNV antibody prevalence in red foxes, striped skunks, feral cats, or badgers. In the raccoons, opossums, and coyotes, exposure to a flavivirus was detected in 69 (33%) of 208 and specific WNV antibody in 45 (66%) of 68. Because of variation in sample quality from carcasses obtained by different means, our results may provide a conservative estimate of the prevalence of WNV antibody in medium-sized Wisconsin wild mammals.

**Table:** Prevalence of West Nile virus (WNV) antibody in medium-sized wild mammals, southern Wisconsin, 2003–2004

Species	No. tested	Virus antibody present	
		Flavivirus, n (%)	WNV specific, n (%)
Raccoons	78	19 (24)	15 (19)
Opossums	71	27 (38)	14 (20)
Coyote	59	22 (37)	16 (27)
Red fox	7	1 (14)	1 (14)
Skunk	6	0	0
Feral cat	5	1 (20)	0
Badger	2	0	0
Total	228	70 (31)	46 (20)

We found similar serologic prevalence to WNV regardless of sex (χ^2^ = 1.329, degrees of freedom [df] = 1, p = 0.26), age (χ^2^ = 1.31, df = 1, p = 0.25), species (χ^2^ = 3.64, df = 2, p = 0.16), or month of collection after September (occurrence of WNV in avian species) (χ^2^ = 1.42, df = 1, p = 0.23). During our sampling period, the prevalence of WNV antibody was 27% (16/59) in coyotes, 20% (14/71) in opossums, and 19% (15/78) in raccoons. WNV antibody was found in 19 (18%) of 105 male animals compared with 26 (25%) of 103 female animals, and in 37 (21%) of 178 adults compared with 9 (30%) of 30 young of the year.

Mosquito transmission of WNV seems most likely in Wisconsin during August through September and less likely after frequent October frosts reduce the general mosquito population. In addition to mosquitoes, WNV may be transmitted by predation or scavenging (*6**,**10*). Previous studies (*3**,**6*), based on small sample sizes, reported relatively high WNV antibody prevalence rates for raccoons (>75%) and opossums (>60%). Our data indicate that the WNV antibody prevalence is similar for raccoons, opossums, and coyotes; however; food preferences differ in these 3 species (*11**–**13*). Raccoons are omnivorous, consuming mostly plant material, invertebrates, and small vertebrates; acorns and other plant materials are important fall food. Opossums are also omnivorous, consuming almost any available animal or plant material; their summer and fall diets consist primarily of invertebrates, small animals, and plant material. Coyotes are primarily predators on small vertebrates and scavengers on carcasses such as deer, livestock, and poultry. Because of the similarities in WNV antibody prevalence and differences in primary food choices, we suspect a common route of WNV transmission, most likely arthropodborne.

A relatively high proportion of medium-sized mammals appear to have been infected with WNV. Whether these species play a role in maintenance and transmission of WNV needs to be determined. Whether raccoons, opossums, and coyotes can be indicators of WNV transmission or potential WNV reservoirs for subsequent transmission to avian, domestic animal, or human hosts is not known. Further research is needed to understand the role these species play in the epidemiology and epizootiology of WNV and the effect of the virus infection on specific populations of free-ranging mammals.
